# Optimisation of a Stirred Bioreactor through the Use of a Novel Holographic Correlation Velocimetry Flow Measurement Technique

**DOI:** 10.1371/journal.pone.0065714

**Published:** 2013-06-11

**Authors:** Mohd-Zulhilmi Ismadi, Simon Higgins, Chaminda R. Samarage, David Paganin, Kerry Hourigan, Andreas Fouras

**Affiliations:** 1 Department of Mechanical and Aerospace Engineering, Monash University, Melbourne, Victoria, Australia; 2 Division of Biological Engineering, Monash University, Melbourne, Victoria, Australia; 3 School of Physics, Monash University, Melbourne, Victoria, Australia; Institute for Frontier Medical Sciences, Kyoto University, Japan

## Abstract

We describe a method for measuring three dimensional (3D) velocity fields of a fluid at high speed, by combining a correlation-based approach with in-line holography. While this method utilizes tracer particles contained within the flow, our method does not require the holographic reconstruction of 3D images. The direct flow reconstruction approach developed here allows for measurements at seeding densities in excess of the allowable levels for techniques based on image or particle reconstruction, thus making it suited for biological flow measurement, such as the flow in bioreactor. We outline the theory behind our method, which we term *Holographic Correlation Velocimetry* (HCV), and subsequently apply it to both synthetic and laboratory data. Moreover, because the system is based on in-line holography, it is very efficient with regard to the use of light, as it does not rely on side scattering. This efficiency could be utilized to create a very high quality system at a modest cost. Alternatively, this efficiency makes the system appropriate for high-speed flows and low exposure times, which is essential for imaging dynamic systems.

## Introduction

The advent of stem cell research has opened a new world in cell therapy and revolutionized regenerative medicine [1], [2], [3], [4], [5] for many degenerative diseases and injuries [6]. Their ability for self-renewal and differentiation to various cell types makes it an effective option as the basic material for various potential treatments by replenishing damaged cells. Furthermore, the possibility to obtain large amounts of these cells, making full use of their self-renewal nature, can make substantial advancement for scientific research and therapy purposes.

Despite the progress in stem cell research in recent years, technical limitations in scaling-up stem cell cultures represent a challenge in stem cell applications. A controlled, reproducible culture system is needed to expand the cells to adequate quantities for successful clinical implementation of stem cells. Cells are commonly grown in a spinner flask bioreactor. This provides a homogeneous culture environment, thus reducing culture variability. Hydrodynamic shear stress is a significant parameter to be considered in a suspension culture bioreactor. High shear could damage the cell membrane whereas low shear could cause agglomeration, which reduces the culture efficiency. In a suspension bioreactor, hydrodynamic shear stress is varied by the agitation rate and the type of impeller. In order to characterize this parameter, a non-intrusive measurement technique is highly desirable to maintain the sterility of the sample. Velocimetry techniques are widely used for various applications. In particular, Particle Image Velocimetry (PIV) is one of the techniques used for characterizing flow profiles.

### Particle Image Velocimetry

There are many qualitative means for investigating fluid flow, including flow visualization methods using smoke, dye, or hydrogen bubbles [7]. To obtain a comprehensive, quantitative understanding of these flows, more sophisticated methods are required. Of these quantitative measurement techniques, the leading three are Hot-wire Anemometry (HWA) [8], Laser Doppler Anemometry (LDA) [9], and Particle Image Velocimetry (PIV) [10]. LDA and HWA are methods that measure discrete points in the fluid volume and may be used in an array or scanned through the flow to record data from throughout the flow volume. This can be a time consuming process and analyzing the discrete data stream from the flow volume may be difficult.

**Figure 1:**
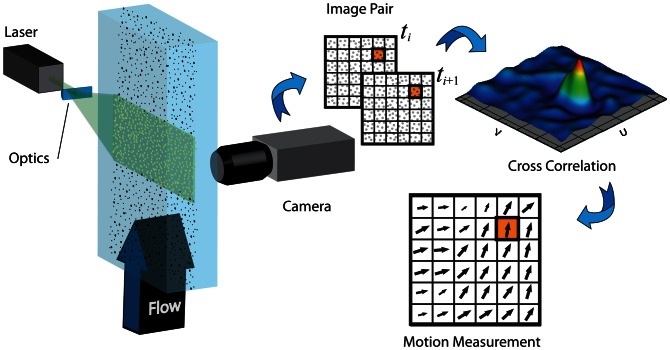
Schematic of conventional 2D PIV. A laser sheet illuminates tracer particles carried by the fluid flow, yielding image sequences captured with a digital camera. The images are discretized into sub-regions and the correlation between subsequent image sub-regions produces a vector of the local particle motion. From this correlation analysis, the fluid motion can be inferred.

PIV is a full field, image based and therefore non-intrusive flow measurement technique that has been gaining popularity over the last two decades [11]. Tracer particles are introduced into the flow, and the region of interest is illuminated using a laser source (typically a pulsed Nd:YAG laser). A very bright light is required because the method relies on inefficient side scatter of light. Figure 1 shows a typical PIV setup. Assuming that the particles faithfully follow the flow, consecutive images of the illuminated region are captured using a high-speed digital camera. The images are discretized into sub-regions and a cross-correlation analysis is performed in each sub-region [12]. The cross-correlation is representative of the probability distribution for the displacement of the underlying particle images within the sub-region, and the maximum signal is the most probable displacement between image frames. Since the time between image frames is known, the velocity of the flow captured in the region can be determined. Processing each of the discretized sub-regions results in a detailed velocity field of the flow. In many cases, it is enough to collect these data from a single plane in the flow. In this traditional form, the method provides no out-of-plane flow information. The most common solution to overcome this is stereoscopic PIV [13], [14], [15]; with two cameras, the local out-of-plane velocity may be calculated. Unfortunately, as laser sheet is the main light source, only a plane of velocity field could be measured at a time. In order to acquire full volumetric flow profile, the position of the light sheet has to be adjusted throughout the volume. Holographic PIV was developed to improve the complex procedure of standard PIV technique in obtaining full field volumetric profile. A number of fully 3D PIV variants have been developed [16] and are briefly described next.

### Holographic PIV

Holographic PIV (HPIV) advanced the methods of recording a 3D instantaneous flow field in experimental measurements of fluid flow [17], [18]. HPIV started with film-based holography, which involves using a reference beam to project the hologram, followed by a 2D plane detector being moved through the projected hologram to record the particle image field. Meng *et al.* [19] looked at the use of film holography and the emergence of direct Digital HPIV. However, in either case, a method to extract velocity data from holographic images is required. This is commonly conducted using two methods, 3D PIV and Particle Tracking Velocimetry (PTV), both of which require reconstruction of 3D images before any inter-frame analysis can be performed [20]. We briefly review each method, in the following two sub-sections.

#### 3D PIV

2D PIV techniques can be readily implemented and adapted for 3D particle fields [21]. In essence, this involves calculation of a spatial 3D cross-correlation using temporally adjacent images, the maximum value of which represents the most common displacement within the 3D sub-region. This requires a highly accurate reconstruction of 3D-images and associated particle images. The reconstructed images often appear exaggerated in the depth direction and this is likely to result in higher ambiguity in the velocity along this axis as described by Pan and Meng [22]. Additions to this method to overcome the loss of accuracy in the depth direction include multi angle in-line holography [20] and multiple off axis holography [23], or a combination of both [17] have been used. However these systems are complex and require accurate calibration and alignment of cameras for corresponding voxel positions [20] and very few groups worldwide utilize this approach.

#### 3D Particle Tracking Velocimetry (PTV)

PTV also requires many individual particles to be reconstructed in space and identified in successive frames in order to track them through the flow. Frequently, the seeding density needs to be drastically reduced in order to obtain images in which particles can be unambiguously identified in 3D space. This is because if particles move in front of or behind one another, the tracking position is lost. With low particle seeding density, collecting data at all regions of the measurement volume is time consuming. To improve this, Pu and Meng [23] derived a Concise Cross Correlation and particle pairing algorithm. This study employed a 3D PIV correlation in discretized volumes, subsequently applying particle tracking to particles within the said volume. The dominant source of error in these techniques is the accurate reconstruction of the particles in the 3D volume.

### Holographic Correlation Velocimetry

In the present study, an alternative approach for volumetric flow measurement is proposed. The proposed holographic technique utilizes a correlation-based analysis to produce the full 3D velocity field without the need for first reconstructing 3D images. This method reduces the amount of processing for digital inline holographic reconstruction and 3D velocity mapping. The method does not use complicated calibration and can be performed with relatively low powered lasers. Due to Mie scattering, forward scattered light is several orders of magnitude brighter compared with side scatter [24]. The efficient use of light with the in-line system allows for high-speed flows to be investigated, which is something highly sought-after in many fields of research. This technique has been titled Holographic Correlation Velocimetry (HCV). This article describes the use of HCV to map the flow in a cuvette as well as the full 3-dimensional field of flow in a conventional bioreactor spinner flask using a tomographic arrangement.

## Methods

### Description of Holographic Correlation Velocimetry

#### Encoding the depth information

**Figure 2:**
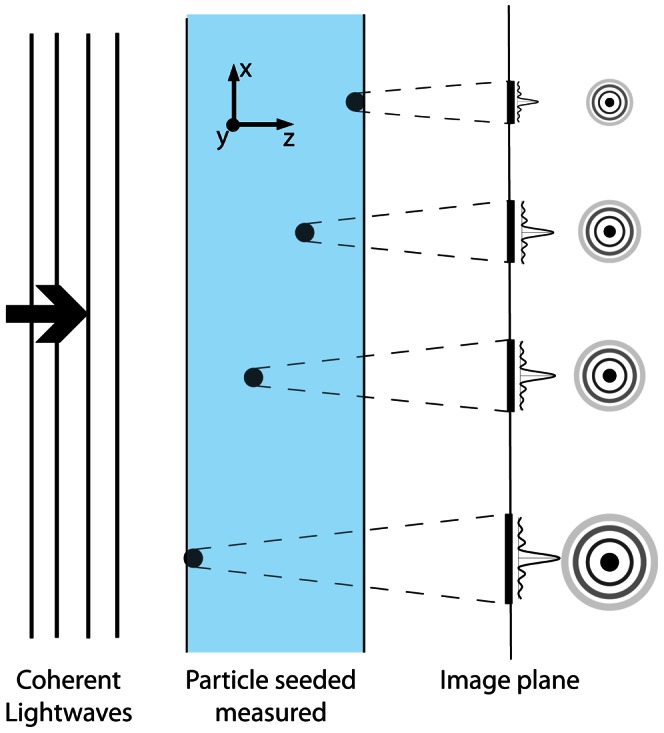
Diffraction pattern of the particle at different image plane distances. As coherent light is scattered by particles, they produce a diffraction image of the particle, which in the far field transversely scales with distance from the image plane. Particle nearer to the image plane produce diffraction rings that are transversely compressed relative to the rings produced by particles further from the image plane. The measured volume gives a speckle pattern comprised of all the overlapping rings.

Unlike PTV, the technique described in this paper is based on a cross-correlation analysis. Fouras *et al.* [25] developed a technique by which the depth information in a seeded flow was encoded by the point spread function of the lens used in cross correlation space to form the images. Cross correlation is a statistical measure of multiple particles, without tracking individual particles. The cross correlation can accurately encode the depth position even in the presence of variable particle diameter. In HCV, the depth information of the particles is encoded by the particle diffraction pattern, embedded in correlation maps, that varies with the propagation distance, *z*. Figure 2 illustrates the concept of how the depth information is encoded. When the Fresnel number, *N_F_ = a^2^/(λz)* – where *a*, is the seed-particle diameter and λ, is the radiation wavelength – is much less than unity, Fraunhofer diffraction is applicable [26]. Given the scaling of the Fraunhofer pattern is in direct proportion to *z*, similar sized particles at different distances from the image plane yield diffraction patterns that are transversely scaled with respect to one another. Hence, the change in the appearance of a given particle’s diffraction pattern is principally due to its distance from the image plane.

This method utilizes the first Born approximation that states that the optical energy density contained in the scattered beam, *Ψ_s_*, must be significantly less than that contained in the unscattered beam, *Ψ_o_*, at each point within the scattering volume: [26].
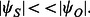
(1)


For this to hold, the ratio of total illuminated area of particles to the total area must be significantly less than 1:
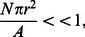
(2)where N is the number of particles, *r* is the radius of the particles and *A* is the total area. Since the number of particles, N, is given by the ratio of the volume of the particles to the volume of a single particle, we obtain the following inequality as a sufficient condition for the applicability of the first Born approximation:
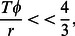
(3)where T and *ϕ* represent the sample thickness and particle volume fraction, respectively. For our situation, we have *T* = 10 mm, *ϕ* = 2.9×10−5 and *r* = 5 µm; this yields T*ϕ*/*r* = 0.058, which clearly satisfies inequality (3). Hence the first Born approximation is applicable to our analysis.

**Figure 3:**
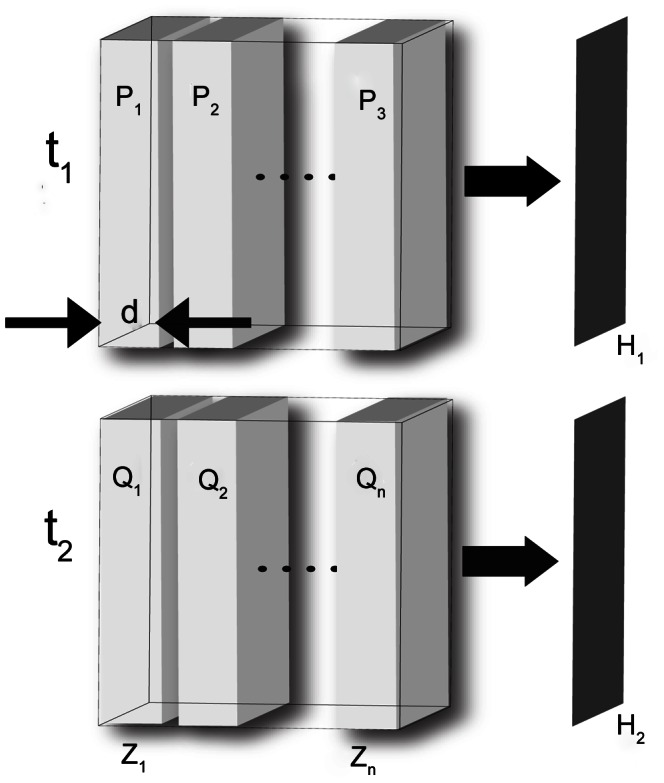
Two holographic images H_1_ and H_2_, at two successive time points, t_1_ and t_2_ for a range of z. *P*_i_ represents the inline hologram due to slice *z*_i_ at *t* = *t*_1_, and Q_i_ is similarly defined at *t* = *t*_2_. Under the assumption of weak scattering by each slab, and neglecting both interference between adjacent particles and an irrelevant additive constant, H_1_ = ∑_i_ *P*_i_ and H_2_ = ∑_i_ *Q*_i_.

Figure 3 shows two inline holograms, H_1_ and H_2_, of a volume at two closely-spaced successive time points, *t*_1_ and *t*_2_, over a range of *z*. *P*_i_ and *Q*_i_ represent sub images of H_1_ and H_2_ that would be formed if only the particles within the slab *z*_i_ were imaged. Under the first Born approximation [27], H_1_ is the sum of all *P*_i_ and H_2_ is the sum of all *Q*_i_. The current method is based on the key assumption that the cross-correlation of the full projected images is equal to the sum of the cross-correlations of sub image pairs. This proposition assumes that the particles are randomly distributed within the measurement volume. This requires that the packing fraction not approach levels that require organized packing or alignment of the particles. A basic proof of this can be formulated as follows with an asterisk (*) denoting a two-dimensional discrete convolution:
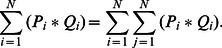
(4)


Since the particles are randomly distributed, there is no inter-particle correlation between adjacent slabs,
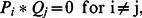
(5)hence,



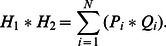
(6)A limitation with current techniques is the particle concentration, whereby high levels of particle seeding can lead to challenges in reconstructing the particle image positions. However, with HCV, a higher level of seeding aids in improving the quality of the cross-correlation that has the depth information readily encoded. This result can be achieved because the spatial relationship is larger than the correlation length.

#### Decoding the depth information

Under the above model for holographically encoding the depth information in the seeded flow, we can formulate an approach by which over a specified depth, we may model a full cross-correlation function for the entire volume.

As customary in PIV, the projected holographic images are discretized into sub-regions. Within each sub-region (which is 2D in the projected image domain), an analytical model of the flow is developed in which the velocity of the flow is specified as a function of *z*. In this case, we have chosen to use an Akima spline [28] as this model. From the known velocity field, the probability distribution function (PDF) is readily calculated. It is well known that the cross-correlation is the convolution of the auto correlation (AC) and the probability distribution function for the displacement [25], [29], [30]:
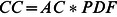
(7)


Since the auto correlation varies with *z*, we invoke equation (7) to give:
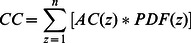
(8)


AC(*z*) can be evaluated analytically, or, in the laboratory by imaging a monolayer of particles at several *z* locations; we therefore have a methodology for constructing the cross-correlation map for any given flow field.

**Figure 4:**
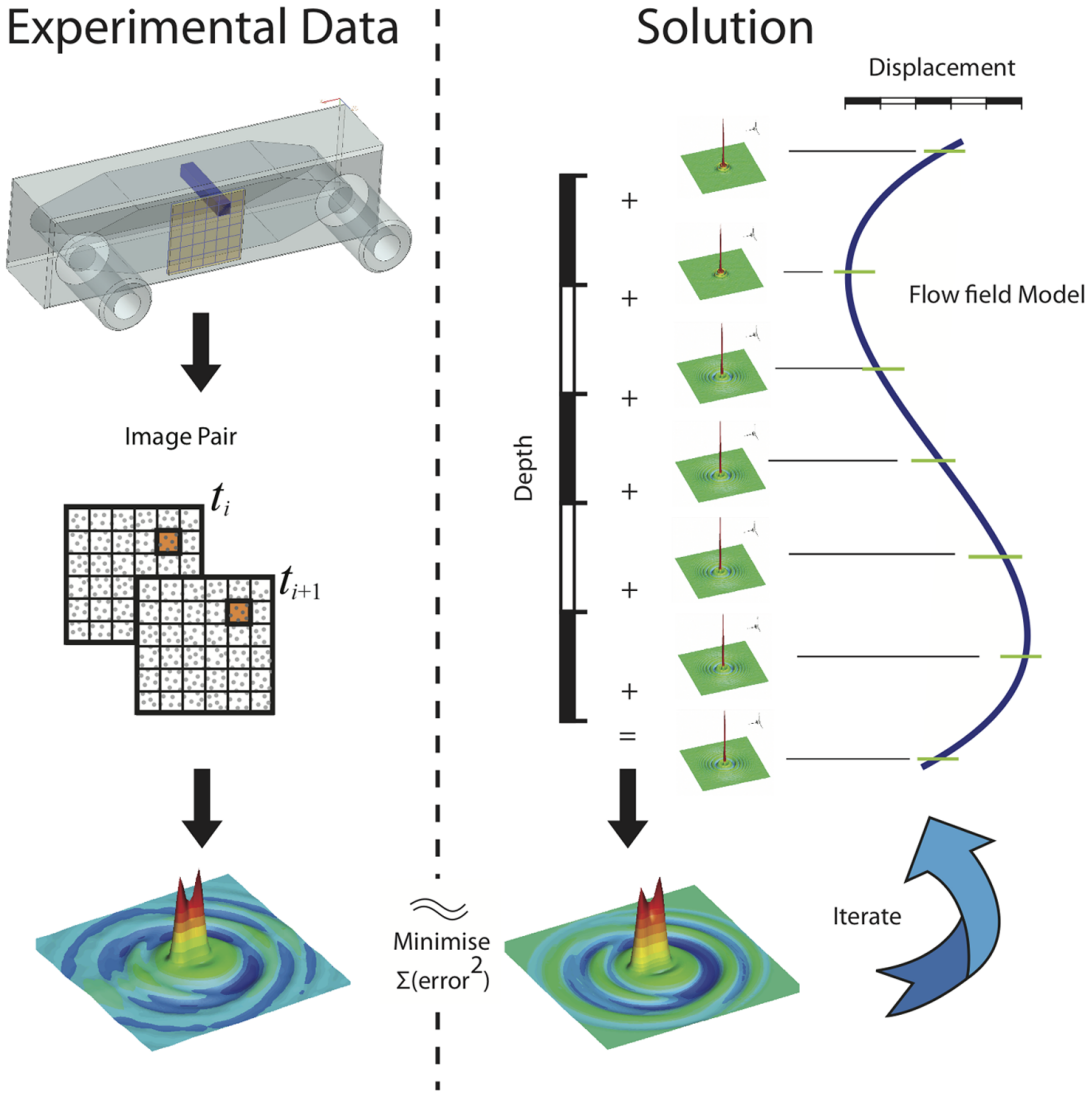
Graphical representation of the Holographic Correlation Velocimetry (HCV) algorithm. The algorithm solves for the 3-dimensional flow field by iteratively reconstructing the volumetric cross-correlation until the residual of the reconstructed cross-correlation and the measured cross-correlation reaches an acceptable level.

Based on this model, we can iteratively reconstruct the 3D velocity field by minimizing the error between the full cross-correlation map from the flow model described above, and the 2D cross-correlation map, obtained for the same sampling region with standard PIV. The modal velocity is measured with standard PIV and contains information that incorporates the velocities of the volume. It is used as the initial velocity estimate for the iterative solver. We use a Levenberg-Marquardt non-linear least-squares solver [19], [20] to perform the error minimization. Figure 4 is an illustration of this iterative process which is implemented in HCV. The Levenberg-Marquardt solver minimizes the residual error between the measured cross-correlation data and the current estimate. The solution is deemed to have converged when the sum-of-squares error changes by less than one part in 10^9^.

Once the in-plane flow has been reconstructed, the out-of-plane flow can be calculated with the assumption that the fluid is incompressible, the volume is fixed, and the flow obeys the law of continuity.

### Modelling

#### Synthetic image generation

To validate the method described in previous section, computer modeling was conducted via generation of synthetic holographic images of particles. These particles are displaced by a known velocity function between successive images. This simulates a flow field of particles in a laboratory fluid flow. There is complete control over the refractive indices and the noise component to the images. The synthetic images are generated with equations from Widjaja & Soontaranon [31] and Tyler & Thompson [32] that are used for holographic particle size analysis and utilize the first Born approximation insofar as they neglect multiple scattering between distinct particles. The parameters for these synthetic images were: 532 nm illumination, 105 mm objective lens, 5000 particles of 10 µm diameter particles per image, and an image of 1024×1024 pixels with pixel size 7.4 µm. The technique uses the diffraction rings, caused by the particles, to record the motion between series of images with depth information included in terms of diffraction. These images are discretized into sub-regions and cross-correlations are generated between successive image pairs. These correlation maps are then decoded in order to determine the velocity through the depth of the discretized sub-regions.

**Figure 5:**
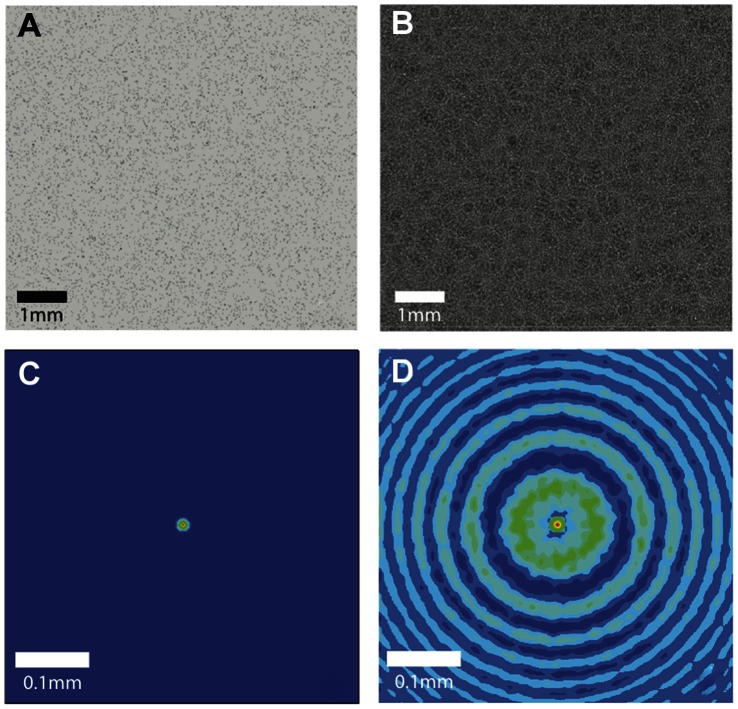
Synthetic image data of particle fields (A, B) and the averaged spatial auto-correlation of the corresponding inline holograms (C, D). The data in A and C represents particles imaged at contact (i.e. *z* = 0), while the particles in B and D are at 20 mm propagation (λ = 532 nm).

**Figure 6:**
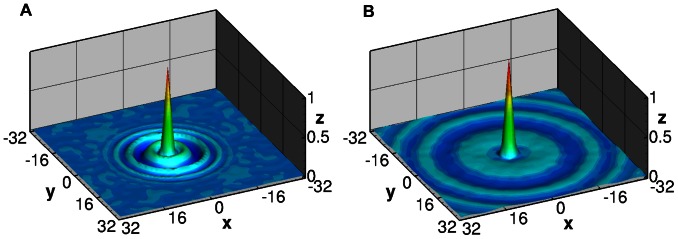
Two, 64×64 pixel normalized, auto-correlation maps from synthetic holographic calibration images of particles having a nominal diameter of 10 µm in a single plane. A) Correlation map corresponds to a propagation distance of 3 mm to the image plane. B) Correlation map at 14 mm propagation distance shows wider diffraction ring pattern. At 0 mm there are no rings visible in the auto correlation. The position and subsequent summation of this calibration map is used to replicate the data correlation maps yielding the velocity direction and intensity at different depths in the flow.

Figure 5 contains synthetically generated images of particle fields (A, B) and the averaged spatial auto-correlation of their corresponding inline holograms (C, D). The data in A and C represent particles imaged at the contact plane (i.e. *z* = 0), while the particles in B and D are at 20 mm propagation distance. Figure 6 shows two example cross-correlation functions of the inline holograms of single-layer particle fields at a small propagation distance and medium propagation distance.

#### Results of synthetic simulation

**Figure 7:**
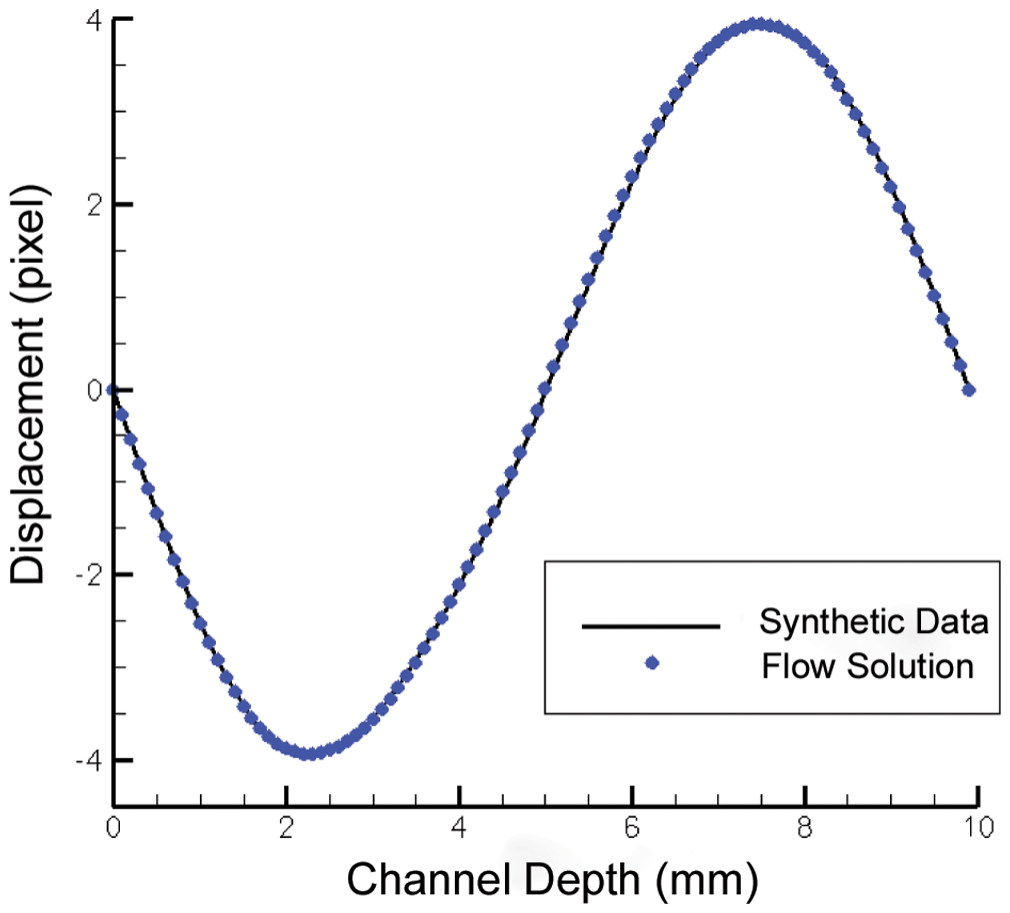
A plot of synthetic flow measurement experiments. The synthetic data (the simulated shear flow between two infinite plates) is shown as a continuous line, with the recovered function indicated by dots. The solution resolved both positive and negative velocity directions from individual cross-correlation maps as illustrated in Figure 8. The normalized RMS difference between the synthetic data and the flow solution is 0.72%.

**Figure 8:**
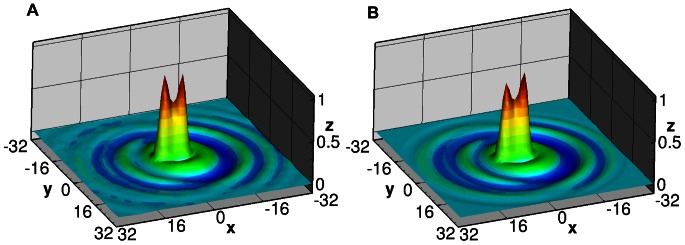
Two, 64×64 pixel normalized, cross-correlation maps for synthetic experiments. A) Cross-correlation of two synthetic image sub-regions. B) Cross-correlation from the combination of calibration maps with the flow model solution.

The results for the synthetic modelling shown in this paper are generated by representing the variation of velocity field in the depth direction with a cubic polynomial. Figure 7 shows the flow input function with 100 data points for the solution fitting function. These data points are the velocity at each of these channel depth locations. Figure 8 gives a side-by-side comparison of a single correlation function from the input synthetic data field and from the output model solution. It can be seen clearly that the model adequately approximates the synthetic data. This flow has been chosen due to its complexity with particles in different depth planes moving in opposite directions, thereby stretching and spreading the correlation map. Nevertheless, the algorithm is able to fit the calibration maps to the data and solve for the correct flow. The initial guess provided to the solver for this experiment was an all-zero or null field. In the simple parabolic case, there may be several similar solutions and hence convergence may be more difficult. But in these cases, this is compensated for by the capacity to supply a good initial approximation for the 3D HCV. This is done from 2D PIV analysis of the same image data sets used for the HCV analysis. With promising results in the synthetic case studies, experimental work was then conducted. The next section outlines the use of HCV for flow in a Flow Cell cuvette (Hellma) as well as the advancement of this method into a tomographic set up in visualizing flow in a thicker sample such as that in a spinner flask bioreactor.

## Experiments and Results

### Flow in Cuvette

#### Experimental set up

A Nd:YLF laser (Darwin λ = 532 nm) was used to generate collimated inline illumination. The laser operates at 40 kHz, which for the purposes of this work can be considered to be continuous wave illumination. This was projected through the Flow Cell (Hellma®137-QS, 10 mm) filled with glycerin seeded with glass particles having a nominal diameter of 10****µm. The particles have a size distribution with 10% having a diameter less than 3.45****µm, 50% less than 9.1****µm and 90% less than 20.31****µm. The holograms of these particles were imaged with a CMOS camera (IDT Y4) with a 200 mm lens (Nikon Corporations, Japan) set with its focal plane 5 mm from the front of the flow cell to allow optimal propagation for the near particles compared to the particles on the far side of the channel. The lens utilized the largest aperture (f-number – f/4.0) to relay light to the CCD. Any alteration in the f-number would alter the required exposure time, as the exposure time is proportional to the square of the f-number. Each image records a volume containing in the order of 2.3×10^4^ particles, which satisfies the first Born approximation.

**Figure 9:**
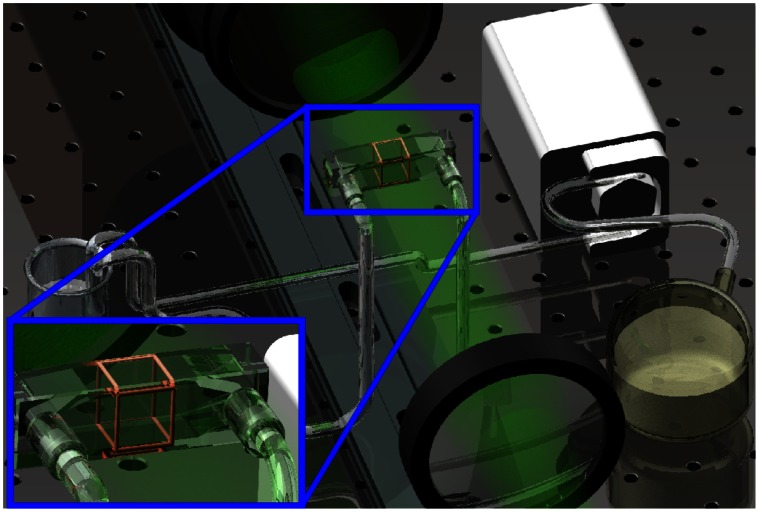
Experimental setup for Holographic Correlation Velocimetry. Shown are the laser, optics, camera, lens, and flow cell attached to the peristaltic pump. A Nd:YLF laser (Darwin 532 nm) was used to generate the inline illumination. This was projected through the Flow Cell (Hellma® 137-QS, 10 mm), which was filled with glycerin mixed with 10****µm glass particles. This seeded flow was imaged with a CMOS camera (IDT Y4) with a 200 mm macro lens (Nikon) set with the focal plane 5 mm from the front of the flow cell for optimized propagation. This flow was maintained with a peristaltic pump through a muffler to remove pulsatility and into an open reservoir. The inset shows the region that is being measured with HCV.

The flow of particles was maintained with a peristaltic pump through a muffler (to remove pulsatility) into an open reservoir as illustrated in Figure 9. The Reynolds number represents the ratio of momentum to viscous forces and is given by *Re = (ρuD)/μ*, where *ρ* is the density, *u* is a representative velocity, *D* is the representative length scale and *μ* is the dynamic viscosity. The Reynolds number of this flow based on the inlet diameter of 2 mm is 1.77. The cross section of the channel is 9 mm×10 mm. The exposure time for the CMOS chip was 762****µs at a 50 Hz frame rate. As the system is based on in-line holography, it is very efficient with regard to the use of light, as it does not rely on side scattering. Most other volumetric measuring systems rely on off-axis or side-scattered light, which requires high power lasers. This efficiency makes the system appropriate for high-speed flows and lower exposure times. Once the images are captured, the analysis is completed on a PC. The calibration images that are used for the algorithm to solve the model for the flow were acquired at 100 depth positions.

#### Data treatment

Images are first filtered to remove stationary artifacts. This background subtraction was performed using the local temporal average of the image sequence. The images are discretized into sub-regions and the cross-correlation map between images frames is computed for each discrete sub-region. These correlation maps have been time averaged as described by Meinhart *et al*. [33]. It has been shown that averaging the correlation maps results in an improved signal to noise ratio by increasing the effective seeding density of the data set [34]. The averaging was performed on the same sub-regions over 400 frames using moving average technique. This system results in a maximum frame rate of 1000 frames per second; this corresponds to 2.5 independent measurements per second. Consider that the dataset consisted of 1000 images; the first average was conducted from image 1 to image 400. Then, another independent average was calculated based on image 401 to image 800. Those averages should be similar as measurements were conducted to steady-state flow. A further increase in temporal resolution is possible by reducing the number of averages, but with the compromise of reduced signal to noise ratio.

This averaging method gives rise to an apparent increase in seeding density. As this is achieved through temporal averaging in the correlation space this does not affect actual seeding density and furthermore the validity of the first Born approximation is still upheld in our analysis.

The image sub-regions of 64×64 pixels were evaluated with a spacing of 16×16 pixels in *x* and *y*. This achieves an overlap of 75%, which has been shown to be optimal [35]. These correlations are produced using a zero padded fast Fourier transform (FFT) function to prevent any wrapping effects over the 64 pixels sub-region of the diffraction fringes from the particles at long propagation distances.

We found that utilizing experimental calibration images introduced noise to the solution. This noise was removed by using synthetic, noise free calibration images generated from first-principle AC maps. This has been implemented in the analysis throughout the rest of this paper. The use of experimental calibration images would be very useful for calibration of flows using poly-dispersed particles or other seeding particles that are harder to model such as red blood cells [36]. The process is simply implemented by placing a sample of the seeding particles in the working fluid on a glass slide that is traversed through the measurement depth.

The 2D PIV results that reveal the modal velocity of the flow field are used to give the first approximation of the solution to the least-squares solver. This allows the algorithm to rapidly converge at an accurate local minimum in what is a very large parameter space.

#### Results

**Figure 10:**
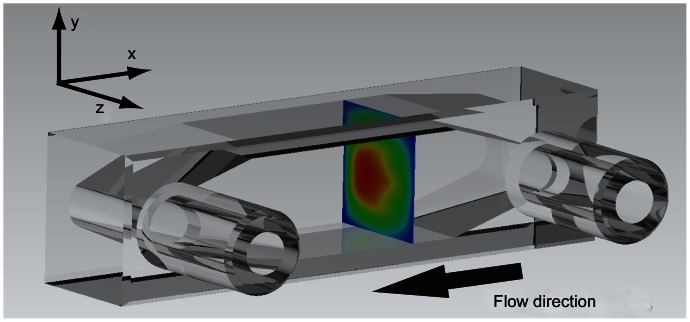
An illustration of the flow channel with the velocity field contour in-situ. The inlet and outlet are shown on the near surface of the (Hellma®) manufactured from fused Quartz (SUPRASIL®). The square cross section of the channel is 9 mm×10 mm ±0.01 mm.

**Figure 11:**
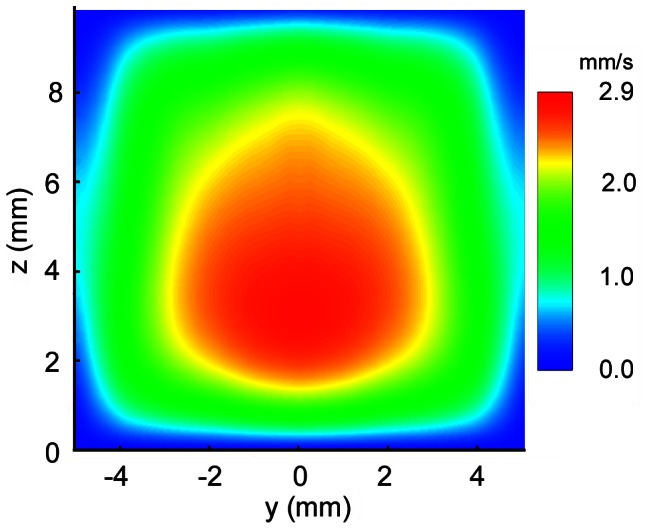
Reconstruction of the out-of-plane velocity magnitude of a single slice of the measured flow within the 9 mm×10 mm flow cell. As the flow enters from the top of the cell (*z* = 10 mm) in a jet like fashion, the momentum carries fluid towards the bottom of the cell (*z* = 0 mm). The peak velocity magnitude is therefore not on the centerline, but closer to the bottom. The flow is symmetric about the centerline at *y* = 0 mm, as expected with the cell geometry.

Figure 10 shows a single slice of the flow in the channel displayed for orientation and context. It is necessary to note that the highest flow rate is toward the back wall on the opposite side of the inlet and outlet ports. The plane shown is towards the inlet port that is in effect acting like a jet into a volume. With this in mind, it can be understood that because the fluid has momentum as it exits the jet, it will be carried to the back wall and then through the channel. Figure 11 shows contours for the magnitude of velocity in a single slice of the reconstructed flow field. On the *z* axis zero is the back wall and we see that the flow is predominantly faster in the lower half of the channel. The flow is extremely symmetrical, which is to be expected from the inlet port being on the centerline of the channel. The corners of the channel clearly illustrate very slow flow occurring here. Again, this is expected in the area where the two walls meet and there is a high drag on the fluid.

**Figure 12:**
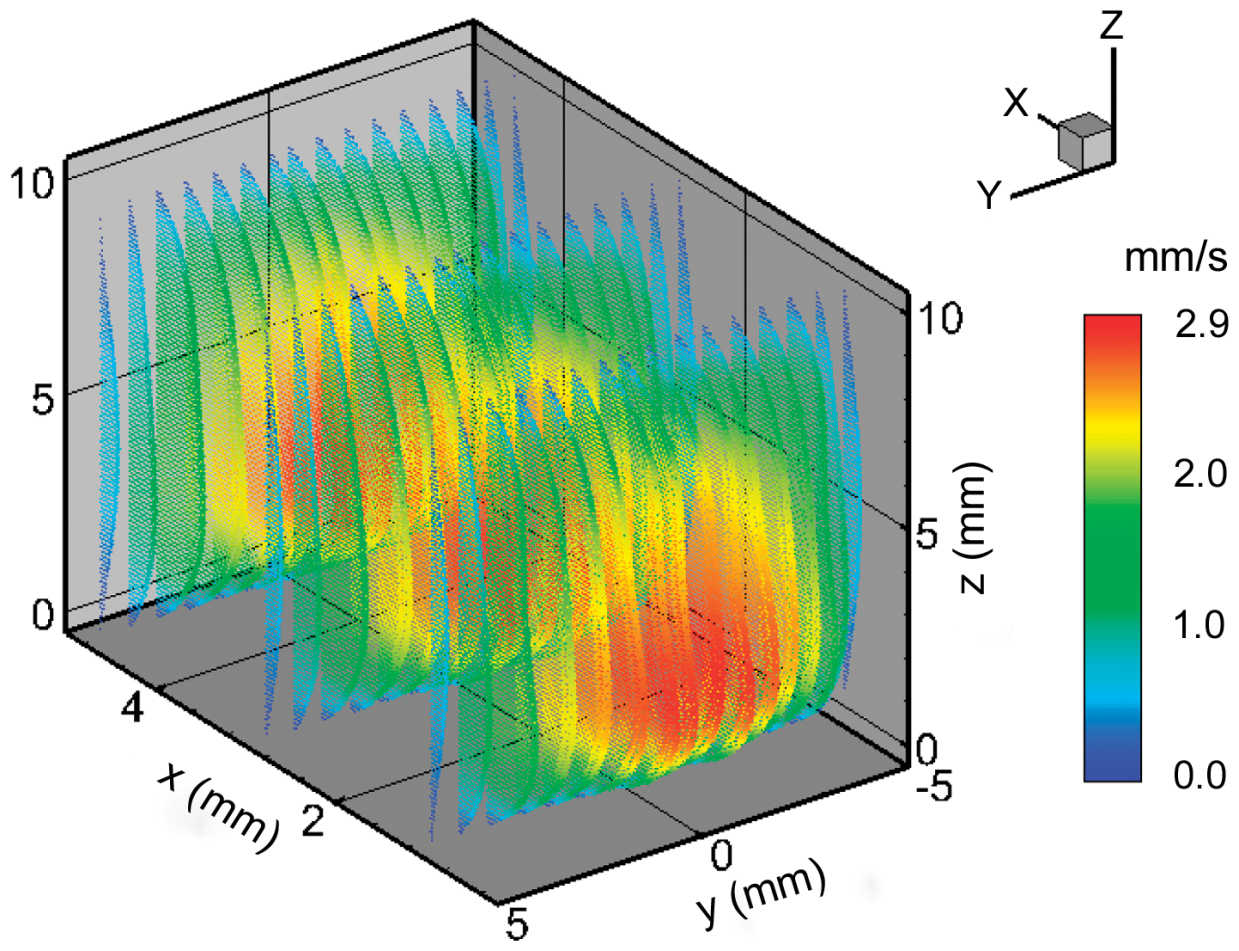
Reconstructed 3D vector field of the fluid flow within the measurement volume. There are approximately 400,000 vectors (colored with velocity magnitude) in the solution. For the sake of clarity, vector resolution has been decreased in *x* and *y*-axes.

To best illustrate that the full 3D volume of the flowing fluid is reconstructed, Figure 12 shows the vectors of over 400,000 points. The spacing between these points is 0.2 mm in the *x* and *y* plane and 0.1 mm in the *z* or depth direction. The vector color is velocity magnitude and there is a single slice of the velocity contour levels. Next, the technique was expanded using tomographic set up to image rotational flow in a bioreactor spinner flask.

### Tomographic- HCV for Flow in a Spinner Flask

#### Experimental set up

**Figure 13:**
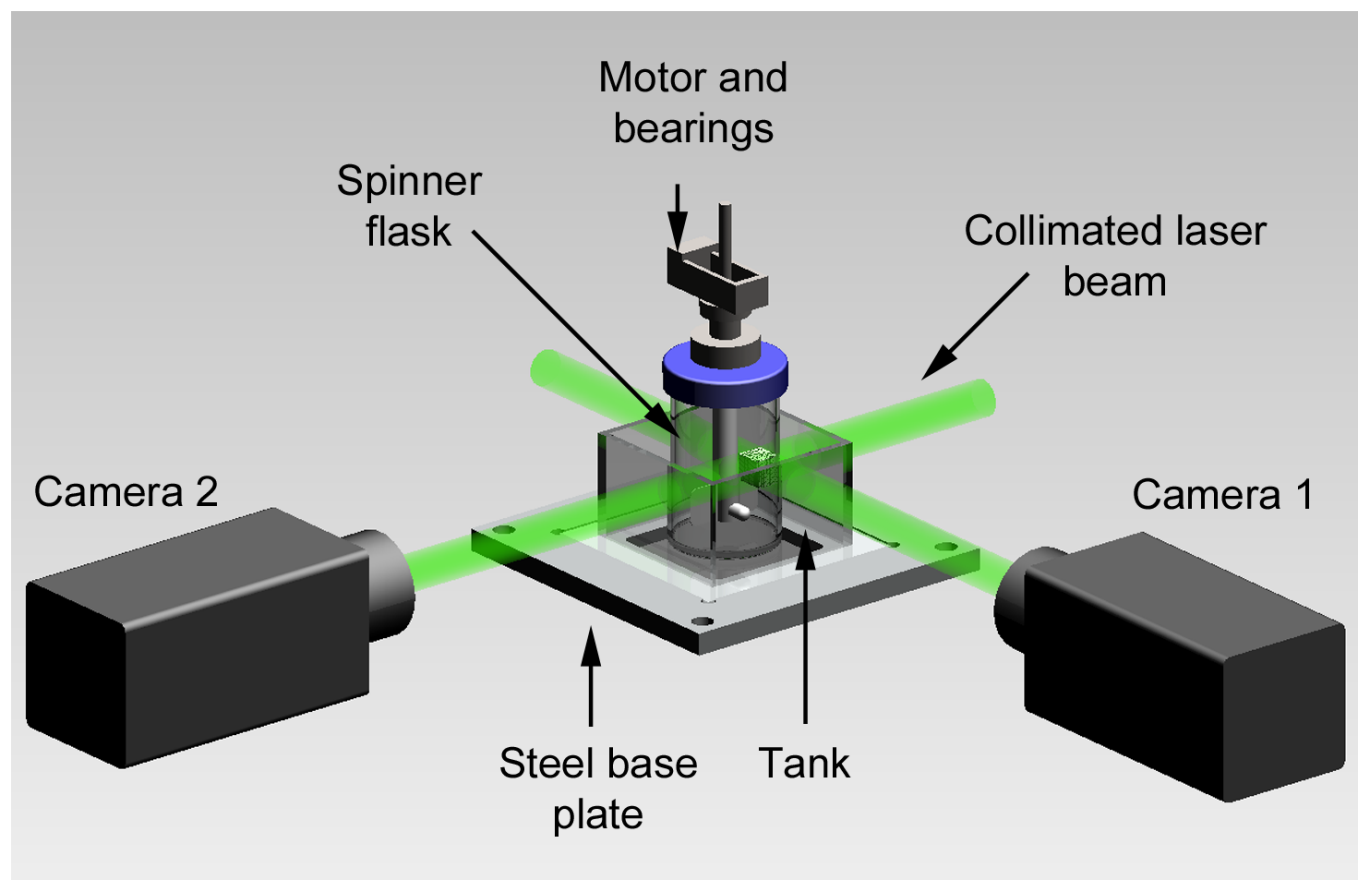
Experimental arrangements for imaging flow in spinner flask bioreactor. The flask was placed inside a rectangular housing filled with water to minimize refraction of laser light. The fluid in the spinner flask was seeded with 10****µm glass particles and stirred by a stepper motor. Two beams illuminated the seeded flow and imaged by two high-speed CMOS cameras (IDT Y4), placed orthogonal to each other to map full 3D velocity profile of the flow.

**Figure 14:**
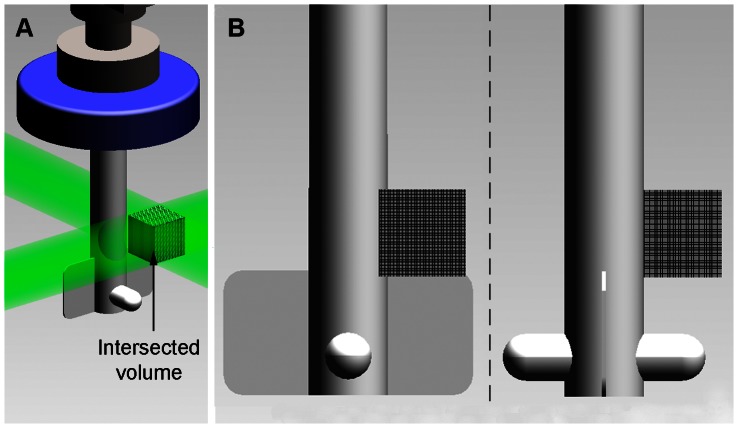
Detailed view of the measured volume. A) Isometric view of the region-of-interest. The intersected volume of the beams is in the size of 11 mm×11 mm×11 mm. B) The beams and the cameras were positioned slightly above the flat impeller shown as the shaded area in the figure.

This experiment used similar laser and optics set up as in the previous experiment. Figure 13 shows the general layout of the experiment. In order to gain full 3D measurement in the system, two orthogonal beams were projected through the 100 mL microcarrier spinner flask (BellCo Glass Inc, USA), having internal diameter 55 mm. The flask was filled with a 100 mL distilled water seeded with glass particles having nominal diameter of 10****µm at 5×10^−4^ g/mL seeding density. The flask was placed inside a rectangular housing, which was also filled with water and has flat exterior faces to prevent the lensing effect due to curved flask surface during the imaging process. The set-up was mounted on a steel base plate and secured to a precision optical table to eliminate any vibrations. The data were recorded with a CMOS camera (IDT Y4) fitted with Nikkor 105 mm f/2.8G lens (Nikon, Japan) at a rate of 450 Hz with exposure time of 54****µs. Due to the nature of the impeller’s opaque material that impeded the field of view, the beam and camera were positioned slightly on top of the impeller for the imaging procedures as shown in Figure 14. Furthermore, each camera captured two consecutive images when the impeller was at a similar position each rotation. 50 pairs of images were taken for averaging purposes.

**Figure 15:**
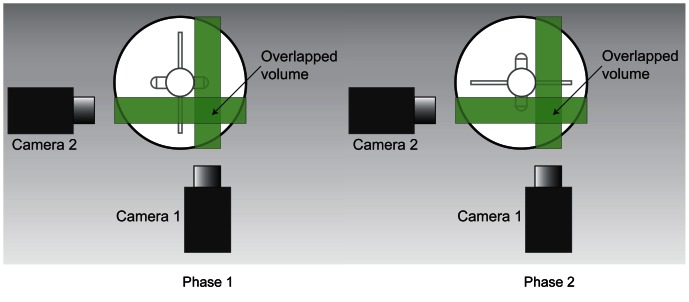
Two phases at which images were captured at each rotation for 50 rpm and 60 rpm as the impeller was spinning in counter-clockwise direction. In Phase 1, consecutive images were captured when the flat paddle was perpendicular to camera 2. On the other hand, Phase 2 captured images when the impeller is 90 degrees out-of-phase to Phase 1.

The flow of particles in the spinner flask was induced with the rotation of the impeller, driven by a stepper motor (Sanyo Denki America Inc, USA) run through a motion controller (National Instruments Australia, North Ryde, NSW, Australia), enabling 5.12 × 10^4^ steps per revolution. The velocity of the motor was further reduced by a factor 30 through the use of a worm gear, which allows a smooth rotation of the disk at all speeds. The Reynolds number of the flow Re = ΩR^2^/ν, ν being the kinematic viscosity (dynamic viscosity, μ per density, ρ) of the water, was based on the radius of the flat impeller (R = 25.3 mm) and rotational velocity, Ω of 5.24 rad/s (50 rpm) or 6.28 rad/s (60 rpm), which translates to Reynolds numbers of 3338 and 4006, respectively. It is important to note that most stirred vessel achieved turbulent condition at Reynolds number higher than 10^4^, having the Reynolds number defined as Re = ΩD^2^/ν [37]. As most fluid mechanics journals used radius as the length scale rather than diameter, the Reynolds number defined in this manuscript is 4 times lower than the Reynolds number utilizing a diameter length scale. The calibration images used in the algorithm to solve the depth of the flow were obtained at 120 depth positions at 32-pixel spacing. Due to the tomographic set-up of the experiment, two sets of data were obtained at one time, one for each camera, from which the overlapped volume was reconstructed to achieve 3D measurement. For each speed, two phases were recorded separately to map the flow in the spinner flask as shown in Figure 15. Flow profiles in front and behind the flat impeller were reconstructed for each spinning rate producing four measurements in total.

#### Data treatment

Each set of data captured by each camera were analysed separately before they were combined to create the volumetric flow profile. Filtering procedures were completed as before. The averaging was performed over 50 pairs of images captured at the same time of each phase. In the analysis, the images were divided into smaller sub-regions in the size of 128 × 128 pixels. The spacing in x and y dimensions was 32 × 32 pixels, allowing 75% overlap as the previous experiment. In this experiment, the calibration images were different than previous experiment. Due to thicker depth of the flask, the calibration images is carried out from 5 mm to 60 mm projection (55 mm thick). The calibration images used the diffraction pattern to solve the depth location (z-axis) of particles in the 2D images from the experiment.

In order to obtain full field 3-dimensional flow patterns, 2 sets of 3D-2-component (3D2C), 90-degree to each other were combined and reconstructed at the overlapped volume. The redundant parameter of each data set (y-direction) was used as a measure to quantify the divergence at every node. Due to the incompressible nature of water, the divergence must be zero. Therefore, in this case, the divergence was a measure of how the values from 2 separate data sets conform to each other.

#### Results

**Figure 16:**
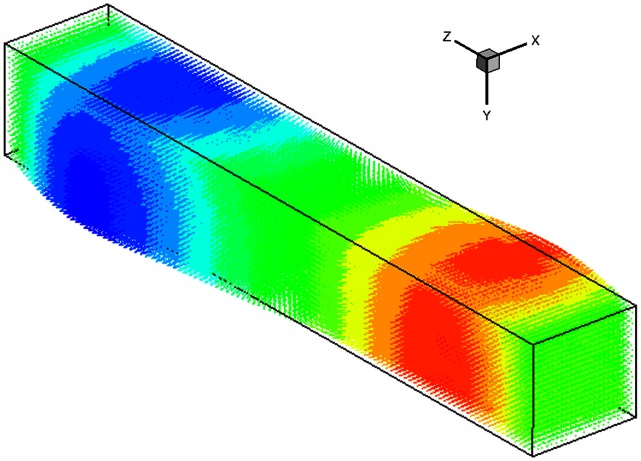
A reconstructed 3D volume (11 mm×11 mm×55 mm), based on images captured with one camera. Two velocity components (u and v) were solved for each camera. At this stage, the velocity along the z-axis (w) was still unsolved. The z-component velocity was acquired from another camera where a full 3D3C vector field can be created at the overlapped volume with reconstruction algorithm.

By having 2 cameras, one camera could capture images in x-y plane while reconstructing the depth in the z-axis, whereas another camera, positioned 90 degrees to the first camera, could capture images in the z-y plane and decode the depth in the x-axis. Figure 16 shows the remodeled volume developed from data gathered in camera 1. It can be seen this configuration is able to capture important features of the flow. As the impeller is spinning in the counter-clockwise direction, the fluid closer to the camera moves in the positive x-direction whereas the fluid further in the depth (z-axis) moves in the negative x-direction.

**Figure 17:**
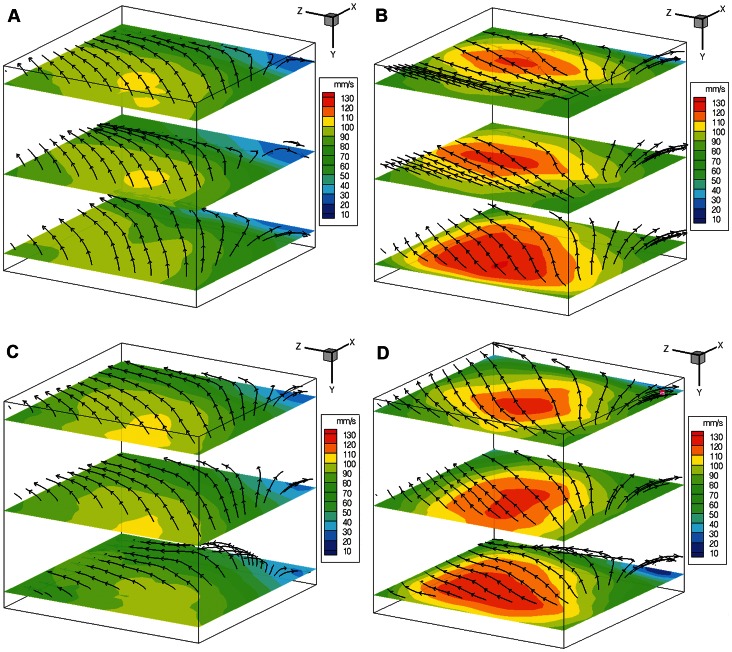
Reconstructed 3D vector field in spinner flask bioreactor in Phase 1 (A, B) and Phase 2 (C, D) regions. A, C) As the flow stirred at 50 rpm (Re = 3338), the intersected volume vector field showed that the flow enters from negative x-plane and exits at positive z-plane. B, D) The fluid enters the volume at higher speed as the flow was agitated at 60 rpm (Re = 4006).

**Figure 18:**
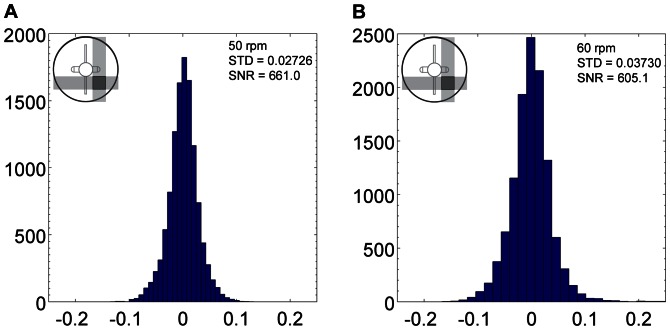
Probability density function of reconstructed volume divergence for flow in spinner flask stirred at 50 rpm (A) and 60 rpm (B) at Phase 1.

**Figure 19:**
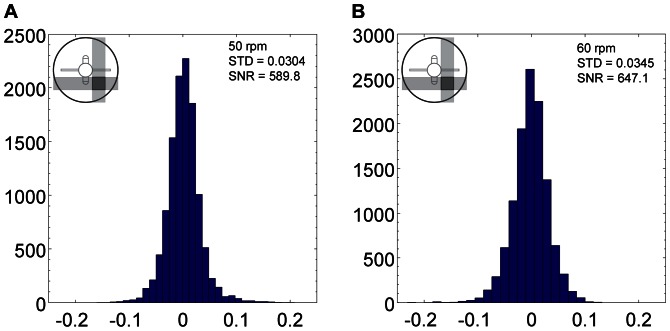
Probability density function of 11638 nodes at Phase 2 region, rotated at 50 rpm (A) and 60 rpm (B).

To create the flow field in the overlapped volume, the start and end of the depth slice for each camera were selected to define the overlapped boundaries of the region. The spacing of layers in the depth has to be similar to the horizontal spacing in order to match each node and ensure accuracy of the reconstructed volume. In this experiment, 32-pixel spacing was chosen in every direction. Based on the captured information, a 3D vector field was mapped, as shown in Figure 17, and the probability density function (PDF) of vector divergence was plotted (Figures 18 and 19).

Figure 17 depicts slices of streamlines and velocity contours of the volume. Some points are omitted in the figure to enhance clarity. The top layer is closer to the free surface whereas the bottom layer is closer to the impeller. As the impeller rotates, it can be seen from the streamlines that the flow rotates and exits at the positive z-plane. In Phase 1, high velocity occurs at the entrance of the volume as the flat paddle impeller pushes the liquid into the imaging region. Lowest velocity occurs near the wall region due to the no slip boundary condition with slight recirculation at the edge. At 60 rpm, the fluid flow had higher velocity with less curvature profile near the free surface. Overall, there was only minor change in the flow topology at this phase.

On the other hand, Phase 2 flow shows some change in topology near the impeller region. The change in height is the trail caused by the flat impeller’s movement. The fluid is forced to flow up and around the flat paddle as it rotates, causing some change in height downstream.

In order to measure the accuracy of the measurement, histograms of the calculated divergence at each node were created for each data set. The standard deviation of the divergence and the signal to noise ratio (SNR) were calculated to determine the statistical variation of the measured divergence and the strength of the proposed technique in measuring the displacement, respectively. SNR is defined by the ratio of maximum magnitude over standard deviation of divergence in the volume as presented in equation (9). Figures 18 and 19 show the divergence histogram for total of 11638 nodes of each volume. All tests show normal distribution profile. The lowest SNR occurred for the 50 rpm data set at Phase 2 with a value of 589.8. The high signal strength in relation to noise demonstrates that the measurement technique is reliable as volumetric velocimetry measurement technique.
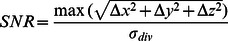
(9)

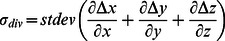
(10)


## Discussion

This study has shown clearly the applicability of HCV in characterizing volumetric flow field. We have demonstrated that the use of HCV can characterize both 2D flow in a cuvette, as well as 3D flow in a spinner flask. This measurement technique is superior to normal stereoscopic PIV, where one layer is measured at one time thus imposing difficulty in the experimental procedure. By decoding the depth based on the diffraction ring in the correlation function, 3D2C measurement field was obtained from 2D images. By having another set of data orthogonal to each other, a full 3D3C flow field was achieved. As the light source is inline with the sensor, a high Reynolds number flow could be characterized due to the efficient use of light. Additionally, as the proposed technique is an optical based measurement, high Reynolds number flow can be characterized by increasing the frame rate at which the camera is acquiring the data. The technique is not only limited to low Reynolds number flow, but rather limited to the maximum acquisition rate of the camera.

This non-intrusive measurement technique presented is suitable for biological flow analysis which sterility is one of the main concerns. Unlike normal flow characterization techniques that involve the use of probes, which have to be immersed in the medium, HCV is an optical-based measurement using a laser and a camera. It allows the medium and cells to be kept isolated in the spinner flask, hence maintaining sterility during the measurement procedure. The volumetric field enables biologists to gain understanding in cell growth and therefore illustrate the important parameters for the proliferation process. The high SNR magnitude proved the reliability in the measurement.

## Conclusion

In this paper, an improved approach for volumetric flow measurement has been developed, in which the correlations of inline holograms can be successfully used to generate a full 3D velocity field of a seeded fluid. It has been shown that these correlations have encoded depth information, providing the velocity at different depths within the fluid. This method allows flow reconstruction without the need to holographically reconstruct the 3D image. Holographic Correlation Velocimetry (HCV) allows for the direct measurement of the velocity field at all depth locations through the use of the volumetric correlation function. Not reconstructing the 3D particle field offers advantages over other HPIV and Digital HPIV systems by directly producing velocity data from 2D images. This approach allows the use of seeding densities in excess of maximum levels for other techniques. Since it does not rely on side scattering, the system makes very efficient use of available light. This efficiency could be utilized to create a high quality system at a modest cost. Alternatively, this efficiency allows low exposure times and the dynamic measurement of high speed flows.

We have shown that this novel technique is able to characterize 3D2C flow in a cuvette and in a spinner flask bioreactor at high signal-to-noise ratio. The laminar flow in a cuvette, with Re = 1.77 showed a maximum velocity of 2.9 mm/s. We have also shown the flexibility of this technique in tomographic set up in the spinner flask experiment. Maximum velocities of 100 mm/s and 130 mm/s were achieved at Reynolds numbers of 3338 and 4006, respectively. The non-intrusive nature of this technique ensures the sterility and suitability for biological flow characterization especially in cell culture procedure. Future work would involve defining the mechanical parameters associated in the process by using this technique. Mixing ability and shear stress are two main considerations that would improve the culture protocol and bioreactor design.
